# Improving Diabetes Management With a Patient Portal: Qualitative Study of a Diabetes Self-Management Portal

**DOI:** 10.2196/jmir.2265

**Published:** 2012-11-30

**Authors:** Sara Urowitz, David Wiljer, Kourtney Dupak, Zachary Kuehner, Kevin Leonard, Emily Lovrics, Peter Picton, Emily Seto, Joe Cafazzo

**Affiliations:** ^1^ELLICSR: Health, Wellness & Cancer Survivorship CentreUniversity Health NetworkToronto, ONCanada; ^2^Canadian Partnership Against CancerToronto, ONCanada; ^3^University Health NetworkToronto, ONCanada; ^4^Health Policy, Management and EvaluationFaculty of MedicineUniversity of TorontoToronto, ONCanada; ^5^Centre for Global eHealth InnovationUniversity Health NetworkToronto, ONCanada

**Keywords:** Diabetes Mellitus Type 2, self care, self management, online, internet, online management portal, patient portal, patient-physician interaction

## Abstract

**Background:**

Effective management and care of diabetes is crucial to reducing associated risks such as heart disease and kidney failure. With increasing access and use of the Internet, online chronic disease management is being explored as a means of providing patients with support and the necessary tools to monitor and manage their disease.

**Objective:**

The objective of our study was to evaluate the experience of patients and providers using an online diabetes management portal for patients.

**Methods:**

Participants were recruited from a large sample population of 887 for a follow-up questionnaire to be completed after 6 months of using the patient portal. Participants were presented with the option to participate in an additional interview and, if the participant agreed, a time and date was scheduled for the interview. A 5-item, open-ended questionnaire was used to capture providers' opinions of the patient portal. Providers included general practitioners (GPs), nurses, nurse practitioners (NPs), dieticians, diabetes educators (DECs), and other clinical staff.

**Results:**

A total of 854 patients were consented for the questionnaire. Seventeen (8 male, 9 female) patients agreed to participate in a telephone interview. Sixty-four health care providers completed the five open-ended questions; however, an average of 48.2 responses were recorded per question. Four major themes were identified and will be discussed in this paper. These themes have been classified as: facilitators of disease management, barriers to portal use, patient-provider communication and relationship, and recommendations for portal improvements.

**Conclusions:**

This qualitative study shows that online chronic disease management portals increase patient access to information and engagement in their health care, but improvements in the portal itself may improve usability and reduce attrition. Furthermore, this study identifies a grey area that exists in the roles that GPs and AHPs should play in the facilitation of online disease management.

## Introduction

Diabetes can be a debilitating chronic disease, with a large number of associated co-morbidities. Effective management requires extensive patient engagement and external support [1,2]. As a result, this population is often studied when exploring innovative chronic disease management tools.

There have been a number of investigations into the ability of Internet-based tools to facilitate diabetes self-management, and some have produced encouraging results [3,4]. Research into the use of diabetes portals has typically shown that access to information and support via online patient-centered tools is capable of improving health indicators for patients and engaging them in the management of their disease [4-6]. Portal use and access have typically been seen favorably by patients, but technological barriers remain (such as with using sophisticated blood glucose monitoring programs). Long-term adherence is the most commonly reported barrier to greater usage of diabetes portals, and findings are mixed in terms of the effectiveness of ongoing follow-up [7]. There is some evidence to suggest that a combination of personalized content, goal setting, and automatic follow-up is effective in keeping patients engaged beyond the short-term [8-11]. Studies on provider acceptance of diabetes portals have shown that providers are often reluctant to adopt these technologies because of lack of knowledge about the Internet or information technology systems [12,13].

Many issues remain unexplored with respect to the scope of online diabetes management portals. Most studies are of Type II diabetics with highly educated, computer-savvy patient participants [3,5-7,14]. Some studies collect “self-management” data, but more commonly previous research has focused on portal use, and success was measured by quantifiable health outcomes (typically changes in HbA1c) [3,6,15,16]. Few studies have sought to assess portal feasibility and usage qualitatively. Those that have do not often consider the health care providers’ experience with the portal, nor do they include the experience of both patients and providers [5,17]. Thus, while Internet-based diabetes management tools have been shown to improve health indicators for diabetics and engage patients in the short term, further research is needed into the scope of such tools and the role played by providers [18].

We conducted a qualitative study to explore the experience of patients and providers with a diabetes management portal implemented by the Waterloo Wellington Local Health Integration Network (WWLHIN).

## Methods

### Patient Portal

The patient portal is an online site that was designed for use by physicians and their patients. Its purpose is to engage patients in self-care and empower them to take a more active role in their diabetes management. The site features disease management tools that allow patients to log health metrics and providers to monitor these patient-entered health metrics, which include blood glucose, blood pressure, and body weight. The portal provides access to two key resources: (1) a “Health Library”, which hosts interactive diabetes education materials for patients and providers, and (2) access to “Personal Health Records”, which is a secure online system that allows patients to consolidate their personal health information. The latter includes contact information, medical and family history, medication details, lifestyle choices, and test results [19], which can be managed by patients and provide them with a comprehensive picture of their health status and health trends.

A patient portal, specifically an online diabetes management tool for patients, was implemented as a pilot program by the Waterloo Wellington Local Health Integration Network (WWLHIN) with patient recruitment lasting for four months. Participants were given an introduction to the portal interface, and technical aspects (such as blood glucose recording) were explained. Based on the severity of the patient’s condition and the self-management needs, participants were stratified into one of three portal use regimens: GREEN, YELLOW, or RED. Participants in the GREEN regimen were asked to record their health metrics 1-2 times per week, those in the YELLOW regimen were asked to record their health metrics 3-6 times per week, and participants stratified into the RED regimen were asked to record their health metrics 7 times a week. Each regimen was tailored by the patient’s clinician to meet the patient’s specific needs (eg, one patient may be required to record their body mass, while another may not); however, it was standard for all patients to record blood glucose measurements at the prescribed frequency.

To evaluate the portal, a six-month follow-up Benefits Evaluation was employed with ethical approval from Institutional Review Board Services, Aurora, Ontario (“HEALTHeCONNECTIONS Project—Benefits Evaluation Program” Version 3.0 dated 2009-06-17). The Benefits Evaluation utilized both survey tools and measures of physiological parameters. Participants were asked to record physiological measures as per the severity of their conditions. Surveys were completed pre- and post-intervention using QuestionPro, an online survey service. Qualitative data collected from this evaluation were used to identify emerging themes to describe the patient and provider experiences with the portal. Results of the surveys are reported elsewhere. This manuscript reports on the results of the qualitatively gathered data.

### Participant Recruitment

Participants were selected from a larger sample population (N = 887) and recruited for the Benefits Evaluation of the patient portal as part of the HEALTH eCONNECTIONS Project. Patients who consented to participate in the Benefits Evaluation were asked to complete a follow-up questionnaire after using the patient portal for a period of six months, and an option for participating in an interview was presented. Those who agreed were considered for an interview. Purposive sampling was used to ensure that respondents to the interviews reflected the demographic of the larger study population. Patients selected by purposive sampling were contacted and, if they agreed to participate, an interview date and time were scheduled. If a patient declined to be interviewed, or could not be reached, alternative patients were selected using the same criteria. This process was carried out until saturation was achieved.

Providers’ responses were gathered through the analysis of responses to five open-ended questions included in a post-study questionnaire. Thematic analysis of responses was conducted, and emergent themes were identified. Providers included a mix of general practitioners (GPs), nurses, nurse practitioners (NPs), dietitians, diabetes educators (DECs), and other clinical staff.

### Data Analysis

Text from transcribed interviews with patients and open-ended responses from providers was coded (by key terms and phrases) and sorted by theme (parent and subtheme). Qualitative data analysis was completed independently by two different members of the research team (EL and ZK). A third member of the team (SU or DW) reviewed all themes and acted as an additional reviewer when consensus was not reached. Emerging themes from the two analyses were compared and contrasted and considered in light of relevant literature. NVivo version 8 software (QSR International, Doncaster, Victoria, Australia) was used to facilitate the coding and sorting process.

## Results

Seventeen patients (eight male, nine female) agreed to participate in semi-structured telephone interviews. There was at least one interviewee from each of the participating WWLHIN Family Health Teams. Sixteen of the 17 patients interviewed were patients with Type II diabetes. The remaining patient had Type I diabetes.

A total of sixty-four health care providers completed the five post-study, open-ended questions regarding their experience with the patient portal. Not all respondents answered each of the 5 open-ended questions. An average of 48 provider responses per question were recorded for the five questions, with a range of 41-58 responses depending on the question.

Four major themes were identified through an analysis of the data, each with several subthemes. The themes were classified as (1) facilitators of disease management, (2) barriers to portal use, (3) patient-provider communication and relationship, and (4) recommendations for portal improvements*.* Below we present each of the four themes together with the subthemes.

### Theme 1: Facilitators of Disease Management

#### Patient Awareness of their Disease

Patient responses generally indicated that the graphs displaying health data, which illustrate significant trends, were very helpful and improved their self-awareness of their health status. They were better able to track their disease. Access to credible health information was said to increase awareness of potential side effects and co-morbidities and often encouraged better disease management.

Provider responses indicated that the concept of the portal was valuable. Providers noticed an improvement in patient awareness and felt that patients perceived they were better managing their disease.

However, providers cautioned that those patients reporting health measures through the portal frequently and consistently may have already been inclined to do so with or without the portal. Providers expressed a perceived concern that too much patient self-care, resulting in a potential for reduced quantity of medical care, was also of some concern. It was occasionally reported that the providers thought some patients would skip necessary appointments or fail to alert a provider of a high blood sugar reading.

#### Access to Information

Analysis revealed that the Health Library was not used extensively by patients. It was thought that this feature could be improved, and it was recommended that information about healthy food options and resources for low-income families should be available. However it was agreed that the information provided was generally viewed positively and thought to be valuable by those that required and accessed it.

Providers also viewed the health information available on the portal positively but felt that accessing the right information at the right time had often proved difficult. Occasionally, the Health Library was not used due to frustration with the portal interface. Some providers who had accessed the library found it cumbersome to navigate.

#### Self-Efficacy and Behavior Change

Patient responses strongly indicated that the portal was used to make small changes to disease self-management behaviors. There was feedback that viewing blood sugar and weight values on the portal alerted them to the fact that they were not adequately controlling their diabetes. As a result, adjustments were made in diet and exercise regimen. Responses indicated that there were patients who felt they were managing their diabetes well and did not feel they needed to make any changes to their self-care regimen.

Providers reported that patients recorded their blood sugar frequently, and there was a perception that viewing trends/graphs had positive outcomes for patients. The reporting and tracking of blood glucose and other health indicators were believed to be the most useful features of the portal for both patients and providers. The portal provided an added source of motivation especially useful for “new diabetics” learning to manage their disease.

**Table 1:** Theme 1—Facilitators of disease management.

Subtheme	Patient quotes	Provider quotes
Patient awareness	“It helped me understand that, so it made me watch my sugar more often when I was in pain. I would check my sugar to find out if it was high or low and try to tie in the highness of the sugar with the pain I was in or you know, stuff like that and with the eyesight as well it took a lot of, like, what I was really worried about was the eyesight when I found I was diabetic and it helped me with that quite a bit...”	“The more that pts [patients] understand their issues the more they are motivated to be responsible…”
		“*Occasionally a patient used the portal to report symptoms that should have triggered an office visit. Also, I am concerned they would use this instead of having their regular formal lab evaluation and follow-up visit”* ^a^
		“They are probably the patients that would bring accurate records to their [appointments] anyway.”
Access to Information	“I found it easy to use and with using the health portal, using that section I found it much easier and faster because it gave me the topics that were relevant to what I was looking for and not a list of suggestions, that might be relevant as well, it just gave me what was relevant to what I was looking for…”	“[The most useful feature was] the Health Library”…
		“*Sharing care could be useful, but it did not seem like mydoctor.ca was utilized as anticipated due to the time it took to use…cumbersome, lots of unnecessary/irrelevant information, little added value…”* ^a^
Self-efficacy and behavior change	“I also found it kind of, you know, embarrassing because I would look on it and say, okay, I haven’t put a blood record in in 52 days and I haven’t really checked my blood, I guess I'd better do that, you know. Like, it gave me the kick in the butt, on the butt to...oh, gee, I better start putting logs again and that.”	“Allowing patients to receive their lab results/data without having to phone in or come in for an appointment. This allows them to receive their health information faster, which may help them be more proactive in their health care.”
		“Patient self management [is the most useful feature], but having someone look over their results for intervention if needed. Best used for new diabetics to help them see patterns, educating as they become used to dealing with their disease.”

^a^ Italicized quotes denote contrasting opinions.

### Theme 2: Barriers to Portal Use

#### Usability and Discoverability

Patients found the patient portal easy to navigate and user-friendly*.* However, improving the convenience of the portal seemed to be important to many patients. Barriers included, but were not limited to, slow dial-up Internet access, the time required to enter data, and the difficulty of data entry.

Providers were generally dissatisfied with the portal’s usability and discoverability (ease with which they could find elements of the portal). When asked what improvements could be made, responses often focused on technical issues. When asked whether they would like to increase or decrease portal use, many who responded *decrease* in portal use cited usability and discoverability as the reasons.

#### Appropriateness

It was often reported that other life events had taken priority over disease self-management and use of the portal. Although not a significant trend, apathy toward the portal and toward disease management in general was occasionally apparent. Some patients felt that they were controlling their diabetes well or found that their health measurements had been fairly stable and therefore did not feel the need to enter information. There was also a tendency for patients to see some information input as trivial or less useful.

Providers believed that accessing patient information was time consuming and sometimes redundant (eg, due to manual data entry). There was often concern that engaging with the portal would decrease the time they could spend with patients. Providers were concerned patients would report health indicators online in addition to calling the clinic office, thereby resulting in a duplicate of provider efforts and a reduction in the quality of care, although there were few reports of this taking place. There were also providers who remained unsure if diabetes was the right chronic disease for the portal as it was believed that this population is already fairly proficient at monitoring their disease.

**Table 2:** Theme 2—Barriers to portal use.

Subthemes	Patient quotes	Provider quotes
Usability and discoverability	“I didn’t enjoy using it. And it was a real pain and it took a lot of time and would rather have had something like, you gave me a blood monitor and I just downloaded it…”	“The problems were largely infrastructure: the program versions; the hardware; the connectivity issues; the actual program for care was fine.”
	“I can’t take a half a day to sit in front of the computer to put the information in.”	“The system is cumbersome and needs an interface that addresses the needs of patients and data entry requirements.”
		“I hate computers and find most interactions frustrating. This is for the future and most of my patients with disease are even less computer knowledgeable than I.”
Appropriateness	“I’ve got other things that are pressing on my mind that I've taken, you know, precedence and overridden everything else that's going on and until those matters get taken care of I've put a lot of stuff that I shouldn't, especially the diabetes and that on the back burner until the other stuff gets taken care of…”	“…many patients brought in sheets of info they felt were important or clinically relevant (eg, individual glucose graphs), which had limited use, but instead served to increase visit time as I educated why the measure was not as important as other indicators. Longer visits, little added benefit.”
	“No, I don’t think so, really. Just ‘cause...I mean...see, I’ve been a diabetic and high blood pressure that has been under control...for a very long time. Well, I know myself probably better than the doctor does, you know what I mean?”	“The messaging system is great yet can be utilized negatively by patients increasing workload on mydoctor.ca and decreasing time for other patient interactions in office. The messaging system has also increased expectations from patients for immediate response.”

### Theme 3: Patient-Provider Communication and Relationship

#### Role of Allied Health Professional

It was shown that patients interacted primarily with an allied health professional (AHP) via the portal. The providers who actively used the portal included dietitians, nurses, nurse practitioners, and diabetes educators. The portal is described as “physician driven”, but it was clear that other health professionals monitored patient health indicators more frequently than physicians.

For those providers that self-identified, it was clear that AHPs interacted with patients more frequently via the portal than family physicians. Although many physicians responded to the questionnaire, they often referred to AHPs in their responses.

#### Provider Engagement Challenging

Although communication seemed to occur primarily with a nurse, dietitian, or other AHP via the portal, patients often wished their physician had taken more of an interest in the program and had reviewed the information they had entered on the portal during their clinic visits (this was also largely done by AHPs). Responses revealed that it would have been beneficial if a health care provider had referred them to information in the Health Library. The responses typically reflected a widespread notion that physicians were often busy and may be unable to fulfill this role as much as they would have liked.

Providers commonly viewed patients’ interactions with the portal positively and their own interaction negatively. Negative comments typically concerned time constraints and technical barriers. There were instances where providers indicated that they believed the portal may be more beneficial for patient self-education than for significant provider usage.

#### Patient Support

When asked, patients generally felt that the portal experience would be improved if there was greater access to clinical support. These patients would have liked to have had a clinician available to explain health information and answer questions that the Health Library could not (eg, about online lab results). Patients often reported reduced anxiety about their health knowing that a health care professional was monitoring their health status. Patients who responded this way also felt reassured knowing they had “access” to their health care professional via the portal at any time of the day. There were also requests for greater ongoing support, as adherence typically declined over the 6-month study period.

It was clear that providers appreciated the ability to view patients’ blood sugar and blood pressure trends, partly because it allowed them to manage patients without in-person appointments and alert them if a health indicator was out of normal range. Some providers expressed concern that patients assumed providers were watching their health status on the portal *all of the time* and might therefore leave problems unreported (ie, some patients assumed that an elevated blood sugar level would be flagged by a health care professional and therefore did not contact their provider).

**Table 3:** Theme 3—Communication and relationship with provider.

Subthemes	Patient quotes	Provider quotes
Role of AHP	“They went over them. [The dietitian] went over [them] when I saw [her] and [the nurse] went through them. [The doctor] never really did go through them… he left it to, like the dietitian and the nurse to go through with me…”	“[The portal] enabled the FHT DM nurse to become better integrated into the communication and care loop with my patients and myself.”
	“Well, the doctors are so busy these days and you really hate to bother them and the nurse was always available.”	“[It was important]…that pts can review their results and can communicate with our diabetic care team; ultimately hopeful that self-management increases and fewer MD visits required”
Provider engagement challenging	“No...I found it easy to use and I guess I would have liked to have seen it more central in my discussions and my appointments with the doctor but that's not a...not a major issue.”	“*Some of this assumes Drs are sitting around with nothing better to do but review volumes of patient lab results”* ^a^
	“Like, I know he checked it once when I was there to see what my records and that were when I was with him and...but that’s just because I was there with them and I believe that even then it came up that it was checked by [the nurse] on the behalf of [the Dr.] not him checking it and I thought that was a little weird. I thought it should be the fact that the doctor actually checked it.	“*[The portal was] somewhat useful for health professional….” * ^a^
		“…*in a busy clinic- we would require to put time aside more to manage patients, which is not actually our mandate in the community.” * ^a^
Patient support	“If I would have had more support from the doctor saying, okay [anonymous], we haven’t heard from you for a while, can you ASAP your information to us so we can keep on contact with you.	“[The portal] allowed timely access to view blood sugar readings entered by patients—I would be able to titrate medications based on values sooner than I would have been able to if having to come in for app’ts.”
	“I really think there should have been more as to what were we expecting our numbers to be? What was appropriate numbers? And where were you at and could you compare your A1C and talk to your doctor every third month because we did this for six months wasn’t it or something…”	“*Some patients take less responsibility in their self-management of the disease as they feel that the health care provider is in constant review of their blood sugars.” * ^a^
	“I felt more comfortable because I knew that somebody was getting my results and they were looking at them and if there was a problem they could email through the portal and just tell me if there’s, you know, you should be doing this or that the other thing.”	

^a^ Italicized quotes denote contrasting opinions.

### Theme 4: Recommendations for Portal Improvements

#### Access to Information

Patients generally responded that the portal content was more than adequate but found it occasionally difficult to access. It was reported that an online tutorial would have been very useful so they could learn and navigate at their own pace. It was difficult for patients to remember the large amount of content taught to them at the portal orientation, and many were not aware of important portal features.

Provider responses revealed that neither they nor the majority of their patients were able to use the portal easily. More training and improved portal usability testing were said to be needed for the portal to be used more effectively. Issues with specific features such as the display of health indicators and with reading weight and exercise values were mentioned less by respondents.

#### Technical Aspect

Patients generally believed that access to information via the portal was easy and more trustworthy than a generic search engine (eg, Google). It was evident, however, that the portal was not used extensively beyond blood sugar reporting and typically the usage declined over time. It emerged that some patients would have appreciated enhanced technical support.

Comments from providers suggested that they often viewed the portal as cumbersome and confusing. Providers commonly responded that communication was slow and the interface difficult to navigate for themselves, other providers, and for patients. However, it is unclear how often these providers interacted with the portal or whether these responses were from GPs or AHPs.

**Table 4:** Theme 4—Portal improvements.

Subthemes	Patient quotes	Provider quotes
Access to information	“[We] were never instructed when we went for our little introduction to doing this, instructed where our numbers should be, what we should do with our numbers, what the heck they were doing…”	“I needed much more orientation, coaching. Nobody seemed to notice I was not engaged.”
Technical aspect	“I did enter my data and in this case I had several months of...I had been collecting data for a num...and I wanted to enter all that data but I had to go back and retroactively enter it and I found that very cumbersome and awkward to do. As soon as I would get a piece of data entered the computer would keep bouncing back to the current date and I had to scroll all the way back again to the next day and enter that data. So, it was very time consuming and awkward.”	“Patients reported that it wasn't always the most user-friendly system. They found the entering of back dates often difficult.”
		“…the technology itself has many little but significant barriers”
		“…cumbersome, redundant, inefficient”
		“If the system was more user-friendly and quicker to navigate it would be more useful.”

## Discussion

In general, patients were satisfied with the features offered by the portal and felt more aware of their health status. However, apart from recording blood sugar readings, patients seemed to use only a small number of features offered by the portal. It is unclear from the results of this study if that is because the features required too much effort or if it was because they were not needed by the patients. It is also important to note that frequency of use varied between patients and often declined over time. Difficulty with fostering long-term adherence is frequently cited in the literature as a barrier to portal use [20]. Portal adherence is difficult to maintain, and the perceived relative value of portals often decreases over time [11,20-22]. This may be because initially patients who use the portal gain an inflated sense of self-management, and therefore no longer view the portal as valuable in their diabetes care [11,22]. The decline in usage may also be explained by the difficulties patients expressed in navigating the portal. Patients reported that an online tutorial would have helped them learn to navigate the portal and its features. This raises concern about the current design of the portal and whether the system was adequately designed for patient use. It is documented in the literature that proper design of information health systems is crucial for maximizing patient adherence and minimizing attrition [9-11,23]. Others have reported similar findings [10]. Russell et al report that, while they could not identify a series of patient characteristics that can be linked to improved engagement in self-management portals, developing a patient-centered, culturally appropriate portal that considers varying levels of health literacy and numeracy may engage a greater number of patients and result in greater overall portal usage [10].

Patients and providers reported a number of small but significant barriers to using the patient portal optimally. These barriers include inadequate ongoing support, poor Internet connections (dial-up versus high-speed Internet), poor orientation, slow data entry, access restrictions (ie, the need to log in with a username and password to view even general health information), and issues with usability and discoverability. Such barriers are common in the literature but have gradually been reduced as more people become familiar with the Internet and high-speed connections begin to reach rural areas [9,24]. Although the results in the literature are mixed [7], usage decline may be overcome by improving access to personalized information, paired with significant ongoing support and improved integration with clinical care [8,9,11,20,25]. Involving patients and providers in the development of a diabetes portal may also be useful in developing a meaningful portal [26].

Patients and providers believed blood sugar reporting was the most useful feature of the portal. This is consistent with the literature, which suggests that diabetes self-management portals see the greatest usage in their blood glucose logging features [10,11]. Research into diabetes management has shown that stricter control of blood sugars leads to reduced health complications, improved quality of life, and may ultimately reduce health care costs [11,22,27-18]. Some studies report that portal users monitoring their blood sugar saw reduced HbA1c levels [6,16]. Patients in our study reported that the portal helped them better monitor their blood sugar; however, information about health behaviors and other health indicators (eg, HbA1c) was not collected. It is also possible that some patients in our study did not realize significant differences in health indicators as a result of portal use. This may be because they became apathetic toward portal use (as suggested by a noted usage decline) and did not realize the benefits of increased blood glucose monitoring over time or their diabetes was already well controlled. This phenomenon has been observed in other studies reporting on the benefits of portal use, where improvements in self-management occurred in patients who are already empowered [11,18], as well as those patients who have access to and a greater understanding of the necessary technology [14]. In this regard, however, the literature is mixed: one study found that patients with a greater perceived need were more engaged and received the most benefit in the use of a self-management portal [11].

Allied health professionals (AHPs) were more active users of the patient portal even though the portal is described as “physician driven”. Provider (often physician) concerns about time constraints (and occasionally, mandate) may indicate that AHPs should act as liaisons between patients and their doctors in portal use, although most patients wished that their attending physician was more involved. The self-management literature suggests that, for patients whose disease is under control, AHPs are the most appropriate health care provider and physicians should be consulted only when the disease is not adequately controlled with self management [29]. In one study, nurses provided the clinical support for users of a diabetes self-management portal exclusively [11]. Few studies examine both patient *and* provider engagement in online diabetes management tools. However, one study found that physicians were hardly engaged at all, while another found that there was no difference in the attitudes of providers [12,18].Involving physicians in the development and design of health portals such as the one analyzed in this paper could enhance their adoption and usage, as the literature suggests that engagement of providers from the inception of health information systems results in greater adherence [30].

Our results suggest that providers generally perceived the portal less favorably than patients. The perception of many of the clinician respondents was that the portal might reduce patient quality of care, as a result of possible missed appointments and a false assumption of provider monitoring of patient entered data. There was also concern that time spent on portal usage would result in less time available for direct patient care. Others have reported additional clinician concerns, including a feeling of “loss of control” as patients become more engaged in their care. As a result, clinicians may be less likely to provide the option of online services to their patients [18]. Although not evident from our results, the communication tools (ie, email) offered by some portals have been identified as another concern for clinicians. Concerns over how to best interpret, document, and respond to patient communications have all been raised. In addition, many current systems do not have a way of verifying the email sender, which raises legal concerns regarding the implications of providing information that may be inaccurate to the sender [14]. Addressing these clinician concerns can help to ensure successful adoption of self-management portals.

### Limitations

Patients who were selected for interviews were representative of the larger study population; however, providers were most likely not representative of the total provider population. It is possible that providers who completed the post-study questionnaire represent a biased sample. Therefore, the generalizability of these results may be limited.

Semi-structured interviews retain internal validity but may lack reliability (a script was used to counter this) and external validity. Open-ended responses retain reliability and some external validity but lack internal validity*.* While the data *collection* methods used in this study complement each other, there are limitations in comparing and contrasting data *types* (ie, interview versus questionnaire *and a* representative population versus a convenience sample).

In addition, the patient portal was available only for 5-10 months (depending on when patients enrolled) and was a pilot test. Patients and providers may have needed even more time to adjust to the portal and may not have been aware of many features due to poor orientation to the system. Provider responses also could have been biased because they may have been commenting on something with which they did not significantly interact (particularly GPs).

Finally, in the instances where this occurred, it was also recognized that limitations exist when making inferences about patients, or providers, based on one’s speculation about the experience of the other.

### Conclusions

The patient portal was shown to be conceptually sound and capable of facilitating patient awareness and perceived empowerment in this population*.*

Patients were mostly satisfied with the services offered by the patient portal and believed it to be a valuable initiative. Provider responses were less favorable (although most believed the concept was good), and some reported concerns that the portal may actually reduce care in some cases.

Frequency of usage was low for many patients and providers, and it was clear that there were small but ultimately significant barriers that either prevented usage or saw its decline over time. Measures are thus needed to keep patients and providers engaged if and when their usage drops. Personalized information, directed ongoing support, and greater involvement in portal design are possible options (although their success has not been proven) to improve patient and provider adoption of any health portal.

This qualitative study of a WWLHIN patient portal shows that such health portals increase patient access to information and consequently improve awareness of, and engagement in, their health care. This study also reveals that defining roles for care (in portal interaction) may be an important next step for diabetes portal development, particularly with respect to the interaction of GPs, AHPs, and patients.

This study contributes to the current body of knowledge on Internet-based health portals, specifically on patient use, perspectives, and health outcomes of these types of portals, and identifies areas in which future research of Internet-based health portals should address. Future qualitative research should thus focus on continued engagement of patients *and* providers in portal development, usage, ongoing patient support and encouragement (clinically and technologically), and the nature of patient-provider interaction via health portals, including mobile applications.
